# Growth factors IGF-1 and KGF and adipose-derived stem cells promote migration and viability of primary human keratinocytes in an *in vitro* wound model

**DOI:** 10.3389/fmed.2025.1516116

**Published:** 2025-02-06

**Authors:** Nina Stadelmann, Raymund E. Horch, Rafael Schmid, David Ostendorf, Ajay Peddi, Theresa Promny, Anja M. Boos, Annika Kengelbach-Weigand

**Affiliations:** Department of Plastic and Hand Surgery and Laboratory for Tissue Engineering and Regenerative Medicine, University Hospital of Erlangen, Friedrich-Alexander-Universität Erlangen-Nürnberg, Erlangen, Germany

**Keywords:** keratinocytes, wound healing, growth factors, adipose-derived stem cells, conditioned medium

## Abstract

**Introduction:**

In the field of plastic surgery, epidermal transplantation is a potential treatment for chronic wounds that results in only minor donor site morbidity. Improving the regenerative capacities of epidermal grafts or single-cell suspensions and therefore accelerating healing processes would be of significant interest.

**Methods:**

In the present study, we analyzed the effects of growth factors and adipose-derived stem cells (ADSCs) on keratinocyte properties. For optimum translation into the clinical setting, primary human keratinocytes and patient-matched ADSCs were isolated and used in an *in vitro* wound model.

**Results:**

The keratinocyte migration and viability increased after treatment with the growth factors insulin-like growth factor 1 (IGF-1) and keratinocyte growth factor (KGF). A similar effect was observed with the use of a concentrated ADSC-conditioned medium (ADSC-CM). It was further possible to isolate the keratinocytes in a xenogen-free medium, which is essential for clinical translation. Importantly, a patient-dependent influence on the effects of the growth factors and ADSC-CM was observed.

**Discussion:**

This study provides potential for the improvement of epidermal transplantation in the treatment of chronic wounds using xenogen-free isolated and cultivated keratinocytes, growth factors, and ADSC. Translating these results into clinical application may help accelerate wound healing and shorten the time until patients can return to everyday life.

## 1 Introduction

In the field of plastic and reconstructive surgery, skin transplantation plays a major role in treating many different diseases. The range of indications includes wounds from burns, trauma, or previous surgery, as well as diabetic or pressure ulcers and autoimmune or vascular diseases. Chronic wounds are especially challenging to treat. They are a burden not only on the patient, with high morbidity and mortality rates (1, 2) and a poorer quality of life (3), but also on the economy due to factors such as insurance costs. Therapeutic approaches are complex, time-consuming, require special medical expertise, and involve high costs (4, 5). Since chronic wounds are linked to age or preconditions such as diabetes or obesity—conditions increasingly prevalent in industrialized countries—approximately 1 to 2% of the population will suffer from a chronic wound at least once during their lifetime (6, 7). There are various approaches to treating chronic wounds, including debridement, hyperbaric therapy, and vacuum-assisted closure therapy, which mainly aim to prepare the wound bed for reconstructive surgery, such as the adaptation of wound edges, flap surgery, or autologous tissue transplantation (8, 9). In chronic and acute larger surface wounds, autologous skin transplantation, full-thickness skin grafts, and split-thickness skin grafts are the most commonly used surgical treatments. Either the epidermis in combination with the dermis in its full thickness or the epidermis with a portion of the dermis in varying thickness are harvested and transplanted. Pure epidermal grafts are less commonly used. Billingham and Reynolds (10) published the use of pure epithelial grafts in 1952, which was later described by Kiistala (11) in 1964. They further reported on the application of epithelial cell suspensions in 1957 (12). The challenge, however, was fixing cell suspensions to the recipient wound bed long enough for adequate attachment and healing. Hunyadi et al. (13) described the use of fibrin to fix epithelial cells to wounds. Experimental and clinical research by Horch et al. (14–16) validated this concept for burn and chronic wounds. In the literature, other keratinocyte carrier materials, such as hyaluronic acid membranes (17), have shown promising results. Pure epithelial grafts have the advantage of creating only minimal donor site morbidity, and it has been suggested that autologous keratinocytes could be the key to closing chronic wounds (18, 19). Recently, an automated device was developed for the simultaneous harvesting of multiple epidermal grafts in a standardized way for clinical use (20, 21). It has been shown that epidermis grafts secrete growth factors, such as vascular endothelial growth factor (VEGF), transforming growth factor alpha (TGF-α), platelet-derived growth factors AA and AB/BB (PDGF AA, PDGF AB/BB), hepatocyte growth factor (HGF), and granulocyte colony-stimulating factor (G-CSF) (20, 22–26). These growth factors can stimulate the migratory behavior of keratinocytes, leading to faster wound closure (27).

The dermal wound healing response is usually divided into partly overlapping phases, including hemostasis, inflammation, proliferation, and dermal remodeling (28, 29). After stopping blood loss and restoring the barrier function by forming a platelet and fibrin clot, the inflammatory response follows to prevent pathogenic infection. This is followed by the proliferative phase, which includes the re-epithelialization process. Activated keratinocytes undergo partial epithelial-mesenchymal transition, changing from their stationary cobblestone-like cell morphology to a flat, motile form (30, 31). Lamellipodial crawling allows keratinocyte migration into the damaged area (32), while keratinocytes behind the migrating epithelium show higher proliferative activity to sustain the cell supply (33). The migratory process is halted by contact inhibition, keratinocytes reattach and readopt their stationary cell morphology once wound coverage is completed (34).

The possible causes for the impairment of wound healing in chronic wounds are manifold, including cellular senescence, excessive inflammation, sustained hyperglycemia and diabetes-associated symptoms, chronic infections, and multifactorial epidermal aberrations (28, 35, 36).

Several proteins might support wound healing. Epidermal growth factor (EGF) is a member of the EGF family secreted by platelets, fibroblasts, and macrophages. It has a paracrine effect on keratinocytes (37). Its ligands bind to the EGF receptor (EGFR) (38), initiating a signaling pathway that can ultimately lead to increased re-epithelialization by promoting keratinocyte migration and proliferation (39–41). Insulin-like growth factor 1 (IGF-1) is a member of the IGF family. It is mainly synthesized by hepatic tissue, though extrahepatic tissues are also able to produce it through autocrine mechanisms (42). Bound to specific binding proteins, it circulates in the blood (43). The proteins IGF-1 and IGF-2 regulate tissue growth, development, and regeneration (44). IGF-1 stimulates keratinocyte re-epithelialization and proliferation (45), even in irradiation-damaged keratinocytes (46), and it is found in high concentrations in cutaneous wounds (47–49). It also leads to wound bed contraction, thus reducing the distance between the wound edges (50). Keratinocyte growth factor (KGF), also known as FGF7, is a protein from the fibroblast growth factor (FGF) family. It is produced by mesenchymal cells and binds to the high-affinity receptor FGFR1-IIIb on epithelial cells, thus exerting a paracrine effect (51). In injured tissue, KGF is highly upregulated (52) and likely promotes the migration, proliferation, and differentiation of various epithelial cells, including epidermal keratinocytes (53–55). Due to its positive impact on wound healing, various therapeutic methods have been evaluated, such as topical application, incorporation into biomaterials-based vehicles, and as a product of transfected cells in gene therapy approaches (56). Thymosins are a family of small proteins originally isolated from the thymus. The most abundant member is Tβ4. Tβ4 is also found in wound fluid (57) and can be released by platelets, which are the first cells to appear in wounds, and cross-linked to fibrin by transglutaminase (factor XIIIa) (58). Tβ4 has been studied in both *in vitro* and *in vivo* models to evaluate its effect on angiogenesis and tissue regeneration. In different models, wound healing could be supported by Tβ4 (59–61).

In addition to growth factors, subcutaneous adipose tissue, such as adipose-derived stem cells (ADSCs), plays a significant role in wound healing (62). ADSCs are multipotent mesenchymal stem cells with the ability to differentiate into adipogenic, chondrogenic, and osteogenic cells (63). They secrete a variety of growth factors that stimulate keratinocyte migration, proliferation, and differentiation, including KGF, EGF, IGF-1, HGF, members of the VEGF family, basic fibroblast growth factor (bFGF), and PDGF BB (64–69). The paracrine effects of the secretome are thought to have a greater impact on tissue regeneration than the ability to replace damaged cells (70, 71). As ADSCs also possess migratory abilities, they are believed to additionally promote wound repair by actively infiltrating the wound (64, 72). Furthermore, they have the ability to modulate transplantation tolerance by suppressing T-cell-mediated responses that cause tissue rejection (73).

The aim of the study was to identify novel approaches enhancing the epidermal wound healing properties of human keratinocytes, which can later be easily implemented into the clinical setting. For this purpose, an *in vitro* model of the human epidermis was established using primary human keratinocytes from different patients to analyze the wound-healing properties of various growth factors such as KGF, EGF, IGF-1, and Tβ4, as well as—as a novel approach—a patient-matched ADSC-conditioned medium (ADSC-CM), with a special emphasis on interpatient differences.

## 2 Materials and methods

### 2.1 Tissue collection from the patients

Human keratinocytes for all experiments, except those conducted in a xenogen-free medium, were isolated from tissue samples obtained from eight patients of both genders (two men and six women) aged between 34 and 48 years (mean age 39.5 ± 8.3 years). These patients had undergone body contouring surgery in the following regions: abdominal (*n* = 6), abdominal and upper thigh (*n* = 1), and upper arm (*n* = 1). Of the patients, five had lost weight preoperatively solely through lifestyle changes, such as diet and exercise, while three had undergone bariatric surgery. The body mass index (BMI) reduction ranged from 11.7 to 43.8 kg/m^2^ (22.6 ± 9.8 kg/m^2^). In all experiments, the number of the patients included was indicated as *n*. Specific information about the patients can be found in Table 1. Symbols were used for data visualization in dot plot graphs.

**Table 1:** Patient information for tissue collection.

Symbols in figures	Tissue origin	Sex	Age (y)	BMI-reduction (kg/m^2^)	T2D	Skin disease	Weight loss
-	Abdomen	M	34	16.8	No	No	Lifestyle
■	Abdomen + upper thighs	W	42	26.7	No	No	Lifestyle
-	Abdomen	W	38	11.7	No	No	Lifestyle
▼	Abdomen	W	46	23.9	No	No	Surgery
▲	Abdomen	W	48	22.1	No	No	Surgery
★	Abdomen	M	48	43.8	No	No	Surgery
♦	Abdomen	W	40	15.6	No	No	Lifestyle
●	Upper arms	W	34	20.3	No	No	Lifestyle

Keratinocytes for the experiments conducted in the xenogen-free medium were isolated from five patients (four women and one men), aged between 43 and 60 years (mean age 54.3 ± 7.9 years), who had undergone body contouring surgery in the abdominal region. One of the patients had lost weight preoperatively solely through lifestyle changes, such as diet and exercise, three had undergone bariatric surgery, and one did not lose any weight before having abdominal tissue surgically removed. The BMI reduction ranged from 0.0 to 49.8 kg/m^2^ (26.2 ± 19.0 kg/m^2^).

Human tissue collection was approved by the Ethics Committee of the Friedrich-Alexander University of Erlangen-Nürnberg (FAU), Germany (Ethics number 264_13B), in accordance with the World Medical Association’s Declaration of Helsinki. Informed consent was obtained from all patients. An overview of the experimental groups can be found in Table 2.

**Table 2:** Overview of the experimental groups.

Cell culture assays	Migration assay	Viability assay	Transmigration assay
Growth factor groups	KGF 1/10/100 ng/mL IGF-1 1/10/100 ng/mL EGF 1/10/100 ng/mL negative control: SRM positive control: KGM (shown in Figure 3A)	KGF 1/10/100 ng/mL IGF-1 1/10/100 ng/mL EGF 1/10/100 ng/mL negative control: SRM positive control: KGM (shown in Figure 5A)	
	KGF 100 ng/mL IGF-1 100 ng/mL KGF 100 ng/mL + IGF-1 100 ng/mL negative control: SRM positive control: KGM (shown in Figure 3B)	KGF 100 ng/mL IGF-1 100 ng/mL KGF 100 ng/mL + IGF-1 100 ng/mL negative control: SRM positive control: KGM (shown in Figure 5B)	
ADSC-CM groups	2-fold concentrated CM 3-fold concentrated CM 2-fold concentrated SRM 3-fold concentrated SRM (shown in Figure 4)	2-fold concentrated CM 3-fold concentrated CM 2-fold concentrated SRM 3-fold concentrated SRM (shown in Figure 6)	
Growth factor groups in a xenogen-free medium		KGF 100 ng/mL + IGF-1 100 ng/mL negative control: reduced EpiLife^™^ medium (rELM) positive control: full ELM (shown in Figure 7)	
Tβ4 groups	Tβ4 0.1/1/10/100/1,000/10,000 ng/mL negative control: SRM positive control: KGM (shown in Figure 8A)	Tβ4 0.1/1/10/100/1,000/10,000 ng/mL negative control: SRM positive control: KGM (shown in Figure 9A)	Tβ4 0.1/1/10/100/1,000/10,000 ng/mL negative control: SRM positive control: KGM (shown in Figure 8B)
		Tβ4 0.01/0.1/100 ng/mL negative control: SRM only supplemented with CaCl₂ positive control: KGM (shown in Figure 9B)	
		Tβ4 0.01/0.1/100 ng/mL negative control: SRM positive control: KGM (shown in Figure 9C)	

### 2.2 Isolation, cell culture, and characterization of the human keratinocytes

Keratinocyte isolation was performed according to the protocol of the “Epidermis Dissociation Kit, human” (Miltenyi Biotec GmbH, Bergisch Gladbach, Germany). In brief, the skin was washed in phosphate-buffered saline (PBS, Sigma-Aldrich Corporation, St. Louis, MO, United States), and subcutaneous fat was removed. The skin pieces of approximately 4 mm in diameter were enzymatically digested using the kit’s enzymes for 18 h. Afterward, the epidermis was peeled off, further digested, and dissociated using a gentleMACS C Tube and a gentleMACS^™^ Octo Dissociator (Miltenyi Biotec GmbH, Bergisch Gladbach, Germany) with running program B. The cells were resuspended in a complete keratinocyte growth medium (KGM) with the following supplements: Bovine pituitary extract (BPE), EGF (recombinant human), insulin (recombinant human), hydrocortisone, epinephrine, transferrin-5 (human), and CaCl₂ (Keratinocyte Growth Medium 2, PromoCell GmbH, Heidelberg, Germany), along with 1% penicillin-streptomycin. The cells were then seeded at approximately 5.0 × 10^6^ cells per 75-cm^2^ cell culture flask coated with 3 μg/cm^2^ rat tail collagen type I (Sigma-Aldrich Corporation) and incubated at 37°C and 5% CO_2_. The medium was changed after 48 h, followed by changes every 2 to 3 days. Antibiotics were omitted after 1 week of cultivation. The keratinocytes were split at a 1:3 ratio using Accutase (Sigma-Aldrich Corporation). After reaching 90% confluence, the keratinocytes from passages 3–6 were used for experiments.

For cell characterization, a monoclonal mouse anti-human cytokeratin antibody (clone MNF116, against cytokeratin 5, 6, 8, 17, and 19; Dako, Agilent Technologies, Inc., Santa Clara, CA, United States) was used. In brief, the cells were fixed using 4% buffered formaldehyde (Carl Roth GmbH + Co. KG, Karlsruhe, Germany), blocked with 5% goat serum (Sigma-Aldrich Corporation), and incubated with the primary antibody (0.64 μg/mL) for 1 h. An appropriate isotype control (Dako) was performed. As a secondary antibody, an Alexa 488 goat anti-mouse antibody (4 μg/mL) (Life Technologies GmbH, Carlsbad, CA, United States) was used. Images were taken using an Olympus IX83 microscope with cellSens (Olympus Corporation, Tokyo, Japan).

### 2.3 Isolation and cell culture of the human ADSCs

Human ADSCs were isolated from the same tissue samples as the keratinocytes. Approximately 30 mL of fat tissue was minced into small pieces of less than 2 mm^3^ and incubated in 0.1% collagenase in PBS (collagenase type I: Biochrom GmbH, Berlin, Germany) at 37°C for 120 min while continuously shaking it on a tube roller. The digestion was stopped by adding 20 mL of minimal essential medium alpha (MEM α) (Gibco^™^, Thermo Fisher Scientific Inc., Waltham, MA, United States) and 10% fetal calf serum (FCS Superior, Biochrom GmbH) and centrifuged at 400 g for 10 min. The top fluid and fat layers were discarded, and the pellet was dissolved in 15 mL of a red blood cell lysis buffer [17 mM TRIS-hydroxymethyl-aminomethane (Sigma-Aldrich Corporation), 16 mM NH_4_Cl (Sigma-Aldrich Corporation)] for 10 min at room temperature. After centrifugation, (300 g, 10 min) the pellet was resuspended in 10 mL of PBS, filtered through a 100-μm cell strainer, and centrifuged again (400 g, 10 min). The cells were resuspended in 10 mL of MEM α with 10% FCS and 1% penicillin–streptomycin, seeded in 75-cm^2^ cell culture flasks, and incubated at 37°C and 5% CO_2_. The medium was changed after 48 h, followed by changes every 2 to 3 days. Antibiotics were omitted after three medium changes. When reaching 80–90% confluence, the ADSCs were split at a 1:3 ratio using Accutase. The ADSCs from passages 3 to 4 were used for the production of a conditioned medium.

### 2.4 Production of the ADSC-conditioned medium

The ADSCs were cultivated in MEM α with 10% FCS at 37°C and 5% CO_2_ until reaching 80–90% confluence. After washing the cells two times with PBS, they were incubated with 10 mL of MEM α without FCS for 24 h. An ADSC-CM was harvested and concentrated at 4,000 g for 30 min using a centrifugal filter device [Amicon Ultra-15 Centrifugal Filter Device (Sigma-Aldrich Corporation)]. The concentrate was dissolved in a keratinocyte basal growth medium supplemented with CaCl_2_, hydrocortisone, transferrin-5, and epinephrine (from Keratinocyte Growth Medium 2 KIT, PromoCell GmbH) to obtain a 3-fold or 2-fold CM, which was stored at −80°C until usage. For control groups, MEM α without FCS was used and treated in the same way as the conditioned medium.

### 2.5 Migration or 2D wound healing assay

Cell migration or 2D wound healing assays were carried out using the OrisT^™^ Cell Migration Assembly Kit (Platypus Technologies, Madison, WI, United States) according to the manufacturer’s instructions. A 96-well plate was coated with 3 μg/cm^2^ rat-tail collagen, and the detection zones were covered using OrisT^™^ Cell Seeding Stoppers. The keratinocytes were seeded in duplicates in KGM in the 96-well plate at a density of 6.0 × 10^4^ cells per well. After 4 h at 37°C and 5% CO_2_, the Cell Seeding Stoppers were removed and the wells were washed two times with PBS to remove unattached cells. Subsequently, the stimulating effect of recombinant human KGF, EGF, and IGF-1 (Biolegend, San Diego, CA, United States) at concentrations of 1 ng/mL, 10 ng/mL, and 100 ng/mL (*n* = 6), a combination of 100 ng/mL KGF and 100 ng/mL IGF (*n* = 6), or Tβ4 [provided by Prof. Dr. Hannappel, Institute for Biochemistry, Friedrich-Alexander University Erlangen-Nürnberg (FAU), Germany] at concentrations of 0.1, 1, 10, 100, 1,000, and 10,000 ng/mL (*n* = 1) in the standardized reduced medium (SRM) (keratinocyte basal growth medium supplemented with CaCl_2_, hydrocortisone, transferrin-5, and epinephrine from the Keratinocyte Growth Medium 2 KIT) was analyzed. As a control, the standardized reduced medium without growth factors was used. As a positive control, KGM was used. The ADSC-CM and respective controls were used at 2- and 3-fold concentrations (*n* = 6).

The cell migration into the detection zones was captured at time points 0 h, 5 h, and 10 h. The uncovered or cell-free area was measured microscopically with one image per well at 40-fold magnification (Olympus IX83, cellSens Software) using Fiji Is Just ImageJ (Fiji, RRID:SCR_002285) 1.51u, an extended distribution of ImageJ. The measurements of the remaining uncovered area in pixels or μm^2^ after 5 h and 10 h were relatively compared to the cell-free area at time point 0, with the latter being defined as 1.

### 2.6 Viability assay

A total of 2,000 keratinocytes per well were seeded in triplicate in 96-well plates with 100 μL of KGM, coated with 3 μg/cm^2^ rat-tail collagen. After incubation for 4 h at 37°C and 5% CO_2_, the medium was replaced with a medium supplemented with either recombinant human KGF, EGF, or IGF-1 (Biolegend, San Diego, CA, United States) at concentrations of 1 ng/mL, 10 ng/mL, and 100 ng/mL (*n* = 6) or a combination of 100 ng/mL KGF and 100 ng/mL IGF (*n* = 5) in the standardized reduced medium. The effect of Tβ4 [provided by Prof. Dr. Hannappel, Institute for Biochemistry, Friedrich-Alexander University Erlangen-Nürnberg (FAU), Germany] at concentrations of 0.1, 1, 10, 100, 1,000, and 10,000 ng/mL was analyzed in the standardized reduced medium (*n* = 3). For further evaluation, Tβ4 (PromoCell GmbH) at concentrations of 0.01, 0.1, and 100 ng/mL was analyzed in either the standardized reduced medium or the keratinocyte basal growth medium supplemented with only CaCl_2_ for comparison (*n* = 2). As a control, the keratinocytes were incubated in the same medium without growth factors. As a positive control, KGM was used. The ADSC-CM and respective control groups were used at 2- and 3-fold concentrations (*n* = 5). At time points 1, 4, and 7 days, 10 μL of a CCVK-I/WST-8 solution (Colorimetric Cell Viability Kit I, PromoCell GmbH) was added to each well and incubated at 37°C for 2 h, protected from light. Cell viability was measured at 450 nm with a reference wavelength of 600 nm (MultiskanTM GO, Thermo Fisher Scientific Inc.). The medium was changed after 2 and 4 days. Absorbance at day 1 was set to 1, and the relative increase in absorbance after 4 and 7 days was calculated.

### 2.7 Transmigration assay

Transmigration assays were carried out using 24-well plates with ThinCert^™^ transwells featuring a pore size of 8 μm (Greiner Bio-One GmbH, Frickenhausen, Germany). After filling the lower chamber with 700 μL of the standardized reduced medium supplemented with Tβ4 (PromoCell GmbH) at concentrations of 0.1, 1, 10, 100, 1,000, and 10,000 ng/mL, 5.0 × 10^4^ cells per well were seeded in duplicates into the upper chamber with the reduced medium without Tβ4 and incubated for 8 h (*n* = 4). As a control, the medium without growth factors was used. As a positive control, KGM was used. The transwells were fixed in ice-cold methanol for 10 min and stained with DAPI (4′,6-diamidino-2-phenylindole, 1 μg/mL, 10 min, Life Technologies GmbH). The transwells were carefully cleaned with a cotton-tipped applicator to remove non-migrated cells from the top of the membrane. DAPI-positive cells were counted manually using Fiji Is Just ImageJ at 4-fold magnification [4 pictures or regions of interest (ROI) per well, one picture per quadrant] (Olympus IX83, cellSens Software).

### 2.8 Viability assay of the keratinocytes directly isolated and cultivated in a non-xenogenic cell culture medium supplemented with the growth factors

The human primary keratinocytes were isolated, as described above. In a collagen-coated 48-well plate (Coating Matrix Kit, recombinant human type-1 collagen, Thermo Fisher Scientific Inc.), 5.0 × 10^4^ keratinocytes per well were seeded in triplicate in an EpiLife^™^ medium (ELM, Thermo Fisher Scientific Inc.) with a reduced concentration of supplements (20% of the regular amount of Supplement S7, Thermo Fisher Scientific Inc.) and the addition of 100 ng/mL IGF-1 and 100 ng/mL KGF at 37°C and 5% CO_2_. As a control, the same medium without growth factors was used. As a positive control, the complete ELM with the regular amount of Supplement S7 was used (*n* = 5). The medium was changed every 2 to 3 days. After reaching 50% confluency, at time points 24, 48, and 72 h, 10 μL of a CCVK-I/WST-8 solution was added to each well and incubated for 2 h, protected from light. Cell viability was measured at 450 nm with a reference wavelength of 600 nm. Absorbance at 24 h was set to 1, and the relative increase in absorbance after 48 and 72 h was calculated.

### 2.9 Statistical analysis

Statistical analysis was performed using GraphPad Prism 8.3.0 (GraphPad Software, La Jolla, CA, United States). Normal distribution was tested with the Shapiro–Wilk test. Differences between the groups were analyzed. In the case of normally distributed data, one-way ANOVA followed by an unpaired *t*-test was used. For non-normally distributed data, the Kruskal–Wallis test and the Mann–Whitney *U* test were applied. Asymptotic significance was used. A *p*-value of ≤0.05 was considered significant. Due to the low number of the patients in the migration assay with Tβ4, no statistical analysis was performed.

Figures show the mean ± standard deviation and were created using GraphPad Prism 8.3.0. Depicted microscopic images were arranged and edited using CorelDRAW X6 (Corel Corporation, Ottawa, ON, Canada).

## 3 Results

### 3.1 Isolation of the keratinocytes and ADSCs

**Figure 1:**
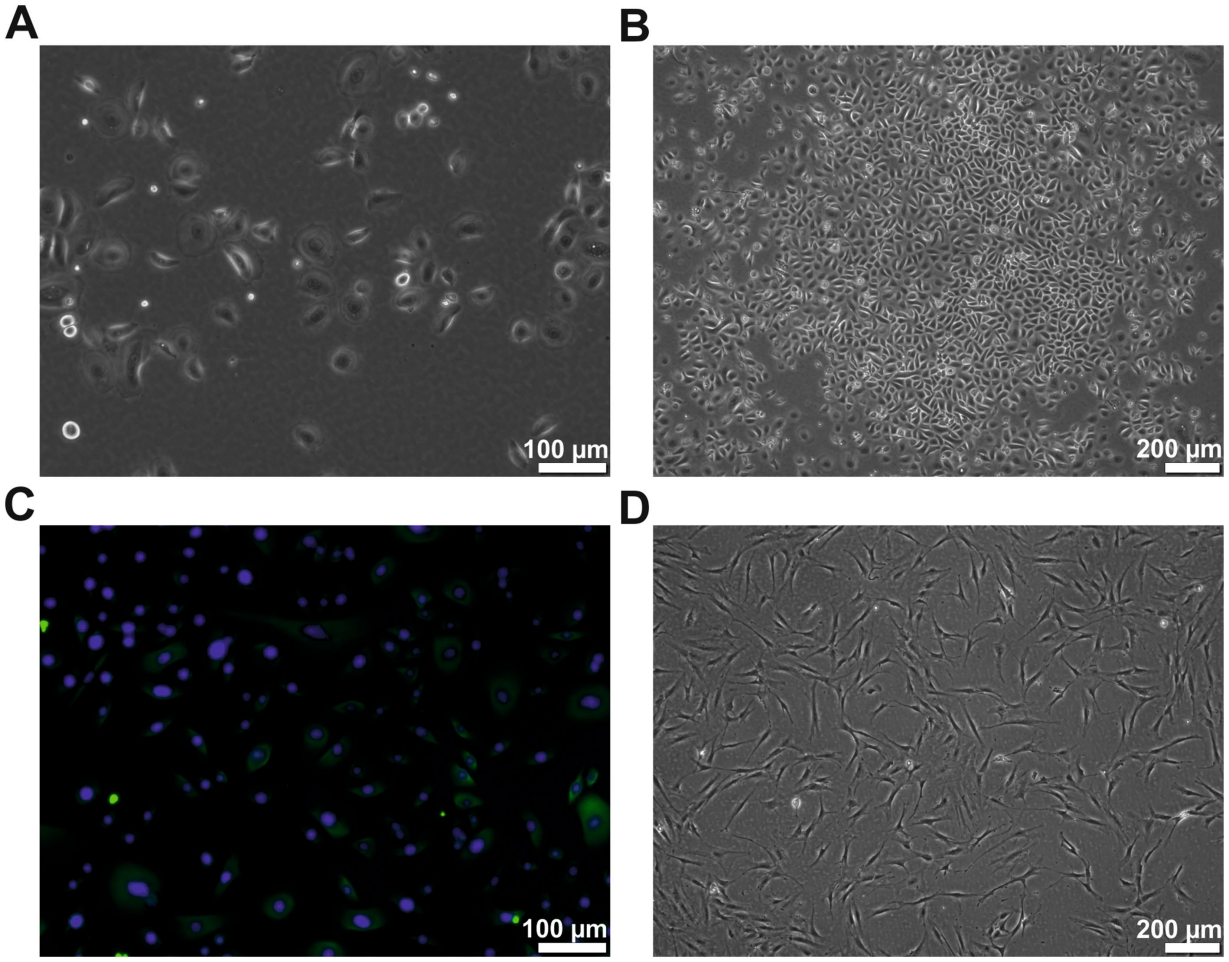
Cell morphology of the human keratinocytes and ADSCs. **(A)** Human keratinocytes at passage 2 showing the typical cobblestone-like morphology. **(B)** Human keratinocytes of passage 2 showing typical colony formation. **(C)** Cytokeratin (green) staining of the passage 2 keratinocytes, counterstained with DAPI (blue). **(D)** ADSCs of passage 2 in typical elongated fibroblast-like morphology. However, few studies have measured the exact amounts of growth factors in a human ADSC conditioned medium. In those studies, the reported quantity of growth factors varied considerably not only from study to study, but also between individual subjects inside the respective studies. Therefore, high interindividual differences in the composition of ADSC conditioned medium is assumed.

Human primary keratinocytes were successfully isolated from all donors. The cells could be cultivated for at least six passages. The keratinocytes showed the typical cobblestone-like morphology (Figures 1A–C) and were cytokeratin-positive (CK 5, 6, 8, 17, and possibly 19) (Figure 1C). The keratinocytes from passages 2–4 were used for all experiments. The patient-matched ADSCs were successfully isolated and cultivated for at least four passages. They showed the typical fibroblast-like elongated cell morphology (Figure 1D). The ADSCs from passages 1–2 were used for all experiments.

Not all assays could be performed with the same number of patients because of insufficient cell numbers. While the effect on the migration and viability of single growth factors could always be performed with the same six patients, the combined growth factor experiments and the ADSC-CM experiments required the inclusion of additional patients.

### 3.2 EGF, IGF-1, and KGF, as well as the ADSC-CM, stimulate the keratinocyte migration in a patient-dependent manner

**Figure 2:**
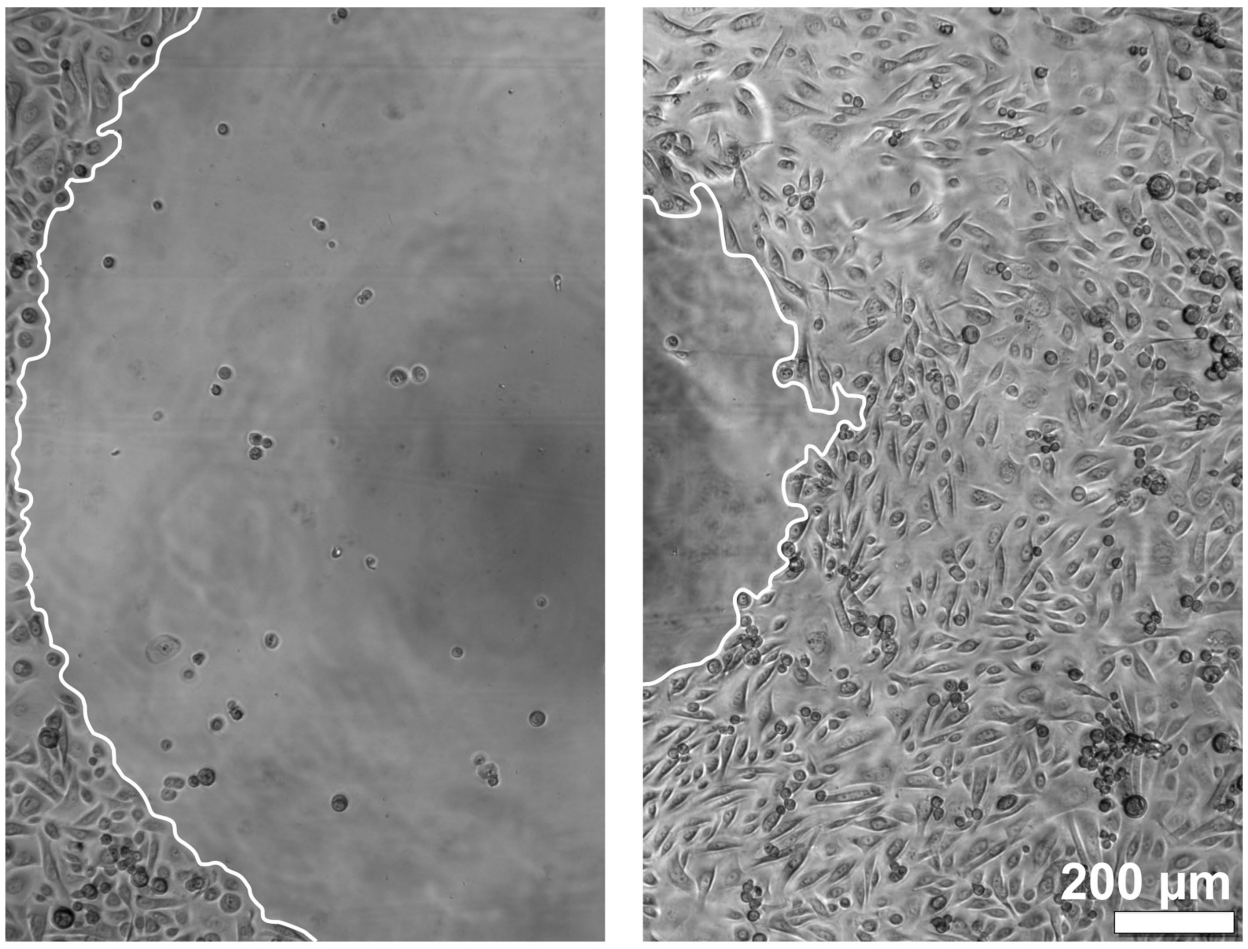
Images of the keratinocyte migration over time. The left image shows the time point at 0 h, and the right image shows the time point at 10 h. The borders of the migration front are marked in white.

**Figure 3:**
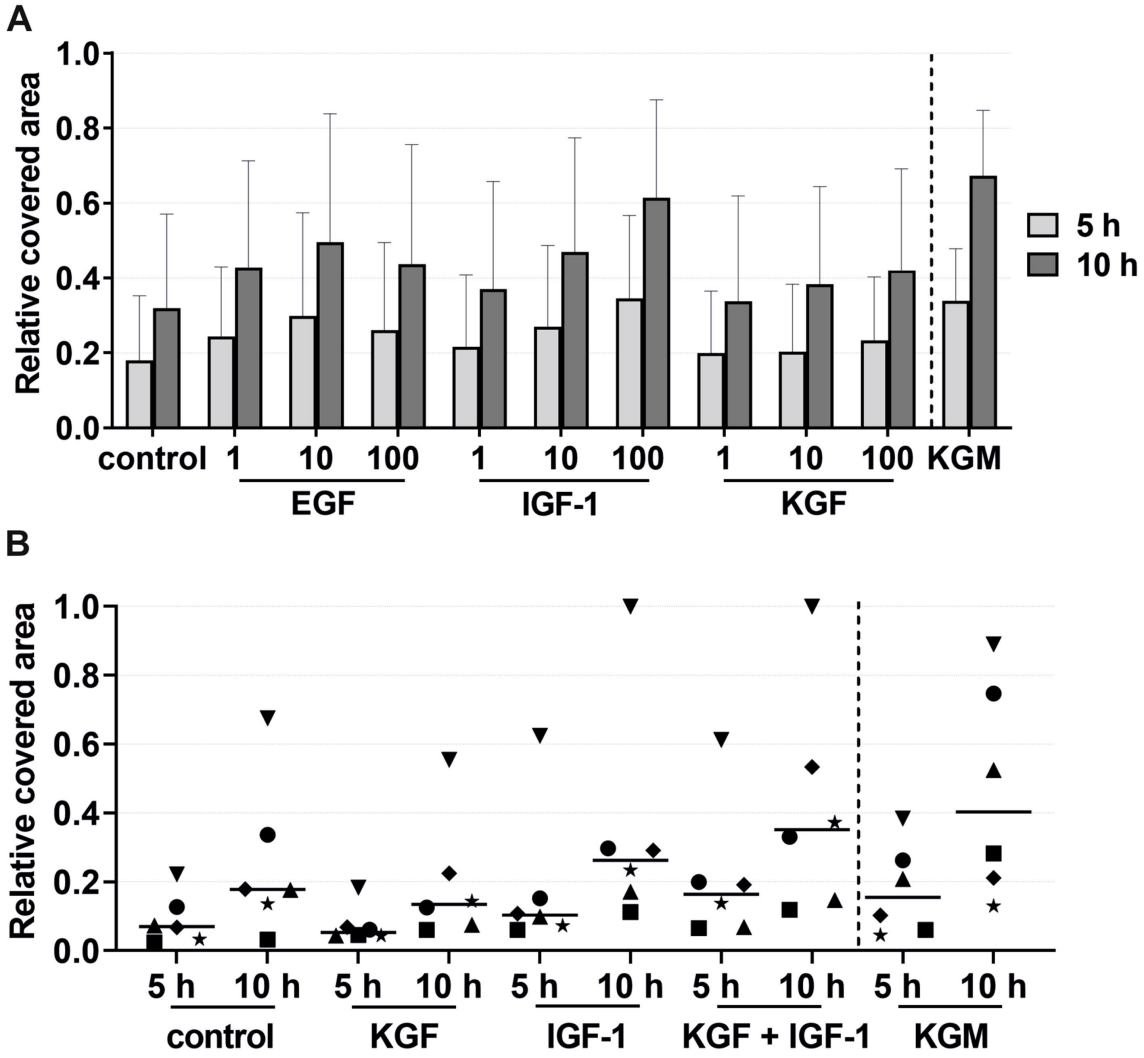
Effects of the growth factors on the keratinocyte migration measured at 5 and 10 h by the relative covered area, with the covered area at 0 h = 0. KGM represents the positive control (*n* = 6). **(A)** Effects of the growth factors EGF, IGF-1, and KGF at concentrations of 1, 10, and 100 ng/mL on the keratinocyte migration. The means are plotted, with error bars indicating standard deviation (*n* = 6). **(B)** Effects of the growth factors KGF and IGF-1 or a combination of KGF and IGF-1 at a concentration of 100 ng/mL on the keratinocyte migration. The values of the individual patients are plotted using different symbols. Horizontal bars represent the mean values of all patients (*n* = 6).

**Figure 4:**
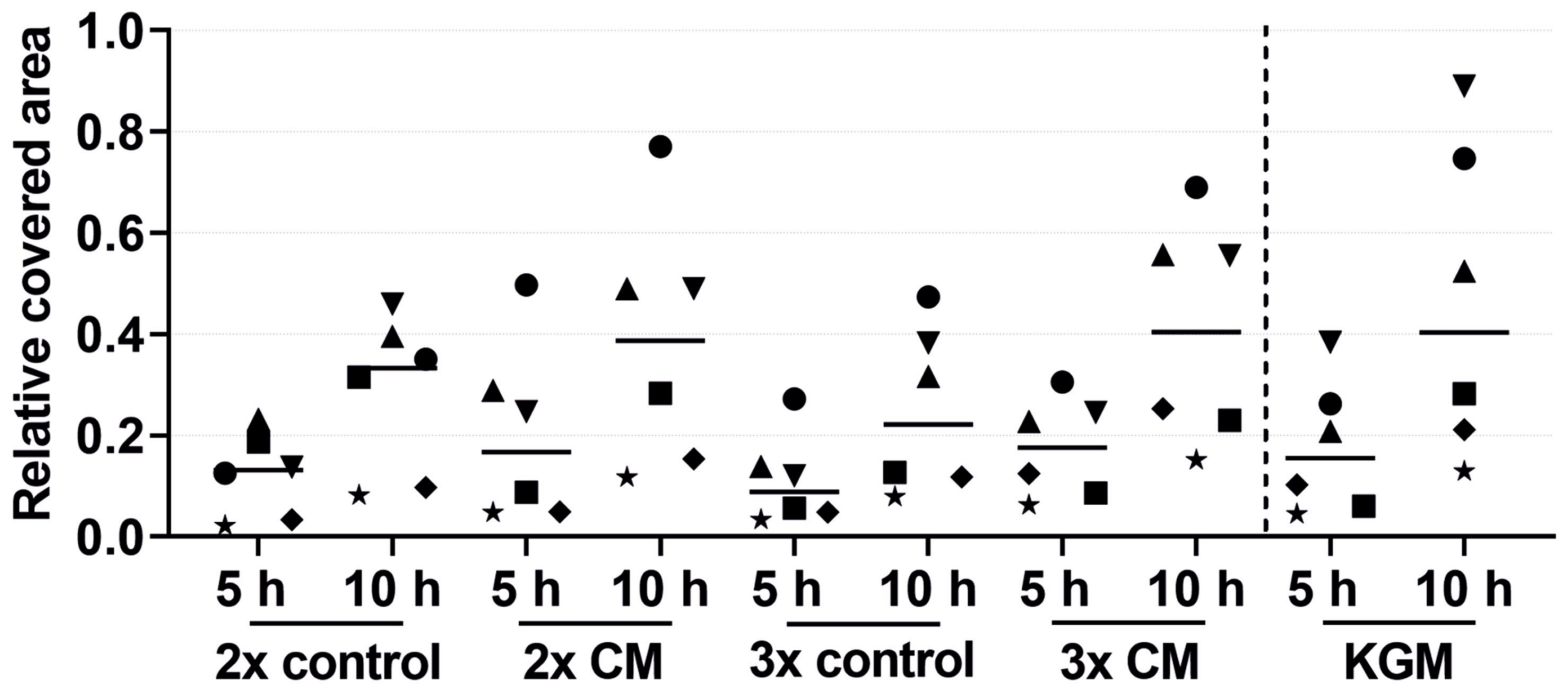
Effects of the 2-fold or 3-fold concentrated ADSC-CM on the keratinocyte migration measured at 5 and 10 h by the relative covered area, with the covered area at 0 h = 0. KGM represents the positive control. The values of the individual patients are plotted using different symbols. Horizontal bars represent the mean values of all patients (*n* = 6).

The migration of the keratinocytes under the stimulation of EGF, IGF-1, and KGF at concentrations of 1, 10, and 100 ng/mL or under the stimulation of the patient-matched 2- and 3-fold ADSC-CM was quantified after 5 and 10 h (Figures 2–4). In all groups, the keratinocytes migrated over time.

In the growth factor groups KGF and IGF-1, there was a tendency for higher migration with increasing growth factor concentrations, although not statistically significant (Figure 3A). In the EGF group, the migration was highest at a concentration of 10 ng/mL. Compared to the negative control group, tendencies were observed for higher keratinocyte migration in all growth factor groups. Combining these growth factors led to an increase, although not statistically significant, in the migratory behavior of the keratinocytes in the majority of the patients compared to the stimulation with a single growth factor or the negative control group (Figure 3B). Interestingly, greater differences were observed between the individual patients. Some patients (marked in Figure 3B as a down-pointing triangle, star, and diamond) showed a significantly higher migration rate in the combined growth factor group compared to the negative group and even the positive control group KGM. In contrast, two patients (marked in Figure 3B as an up-pointing triangle and circle) showed a relatively low response to the combined stimulation.

The keratinocytes cultivated with the patient-matched ADSC-CM showed a tendency for a higher migration rate in both the 2-fold and 3-fold concentrated CM groups compared to the negative control group (Figure 4). The keratinocyte migration in the 2- and 3-fold concentrated CM groups was similar at time points 5 h and 10 h to that in the positive control group. Despite the visible effect, there was no statistical difference. Likewise, as in the combined growth factor groups, high-responder (marked in Figure 4 as an up-pointing triangle, down-pointing triangle, and circle) and low-responder (marked in Figure 4 as a square, diamond, and star) patients were observed, with the high responding group showing above-average stimulation and the low responding group showing below-average stimulation. Comparing the average values of the 2- and 3-fold concentrated CM groups, no differences in the migratory behavior were detected.

### 3.3 EGF, IGF-1, and KGF, as well as the ADSC-CM, stimulate the keratinocyte viability

**Figure 5:**
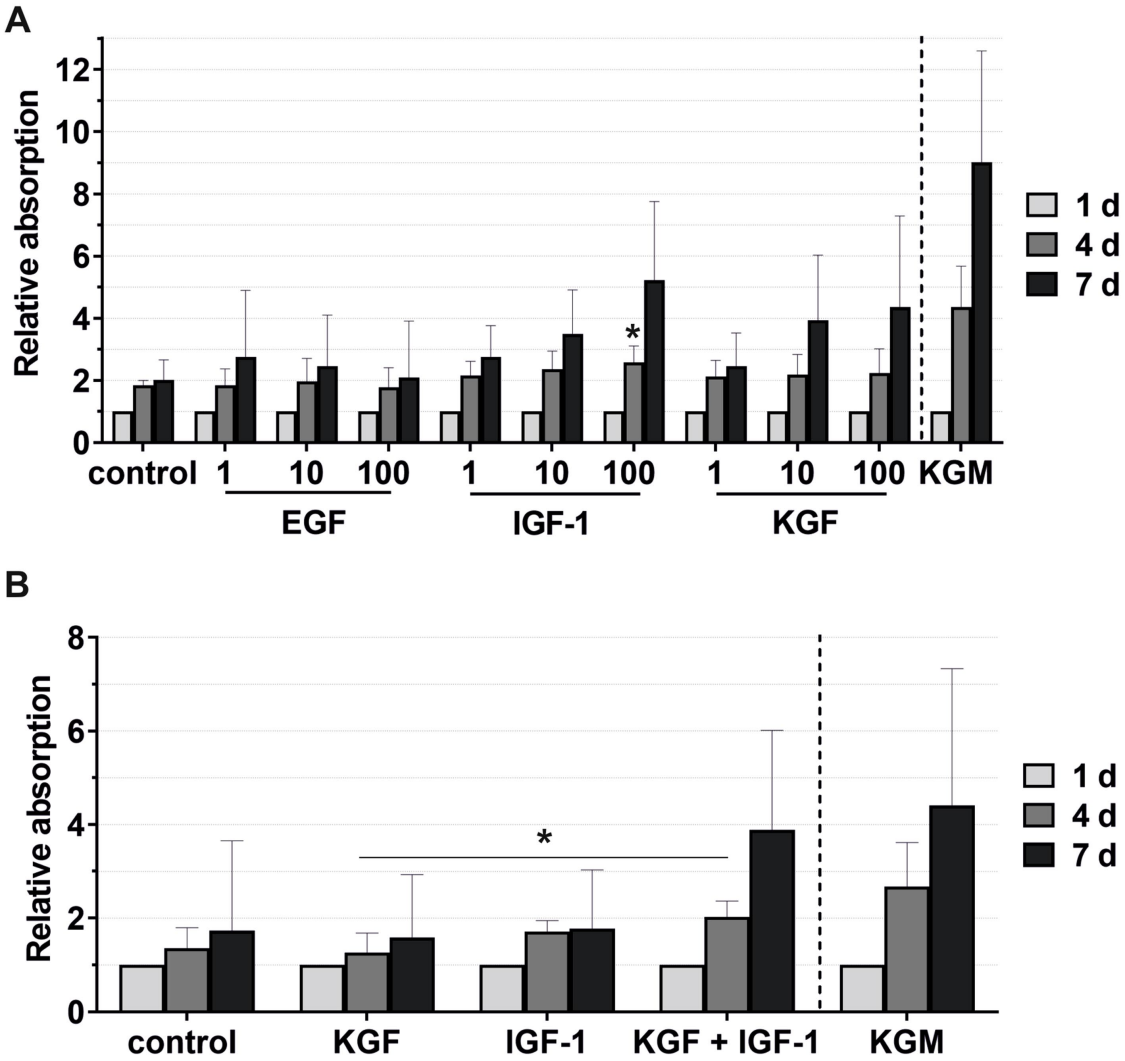
Effects of the growth factors on the keratinocyte viability measured at 1, 4, and 7 days, with the absorbance at day 1 set to 1. KGM represents the positive control. **(A)** Effects of the growth factors EGF, IGF-1, and KGF at concentrations of 1, 10, and 100 ng/mL on the keratinocyte viability. The mean values are plotted, with error bars indicating standard deviation (*n* = 6). **(B)** Effects of the growth factors KGF and IGF-1 or a combination KGF and IGF-1 at concentrations of 100 ng/mL on the keratinocyte viability. The mean values are plotted, with error bars indicating standard deviation (*n* = 5). ^*^p ≤ 0.05.

**Figure 6:**
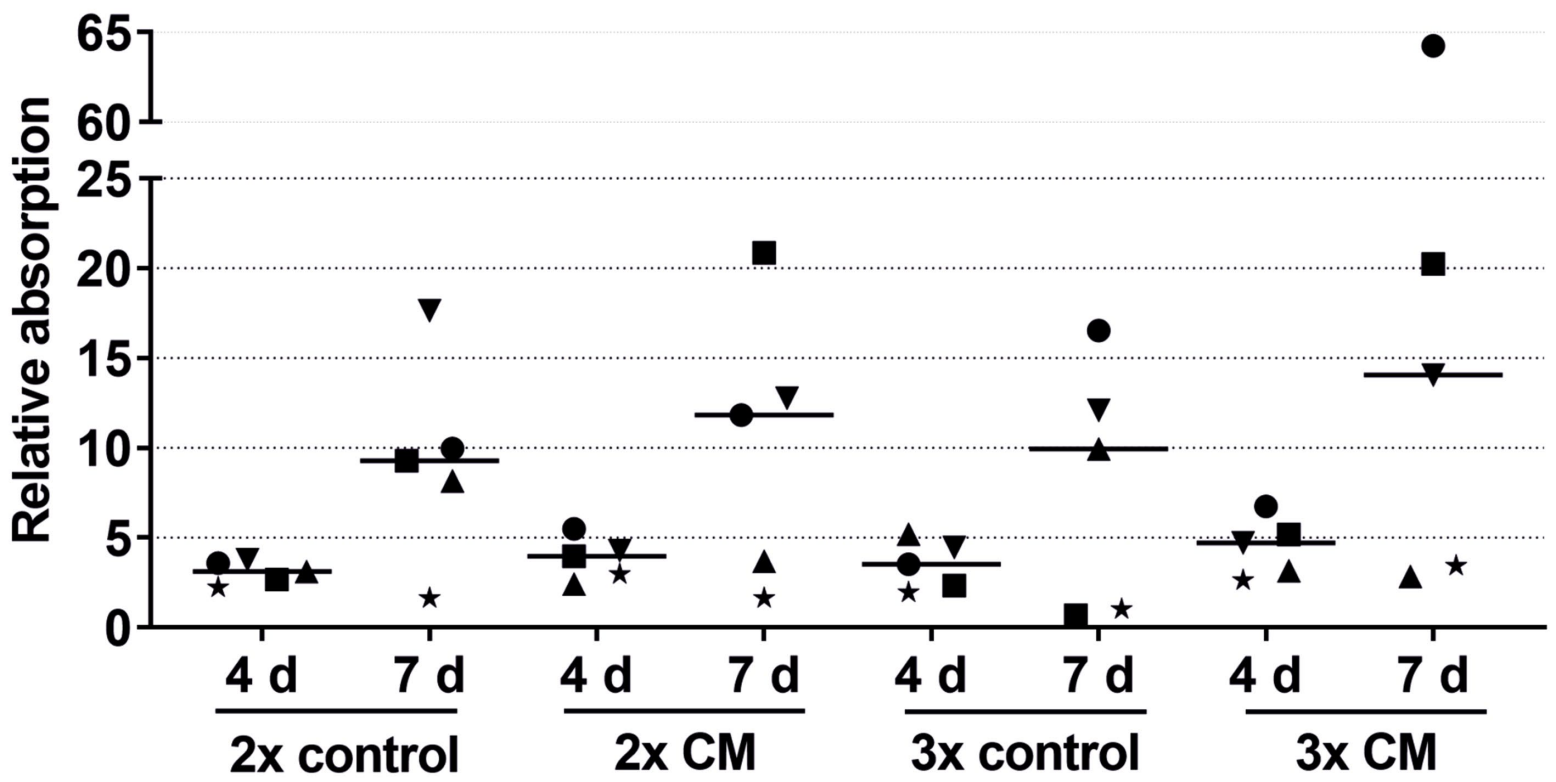
Effects of the 2-fold or 3-fold concentrated ADSC-CM on the keratinocyte viability measured at 1, 4, and 7 days with the absorbance at day 1 set to 1. The values of the individual patients are plotted using different symbols. Horizontal bars represent the mean values of all patients (*n* = 5).

The viability of the keratinocytes under the stimulation of EGF, IGF-1, and KGF at concentrations of 1, 10, and 100 ng/mL or under the stimulation of the 2- or 3-fold ADSC-CM was quantified after 1, 4, and 7 days (Figures 5, 6). In all groups, the keratinocytes showed increasing viability over time. In the growth factor groups, KGF and IGF-1, there was a tendency for higher viability with increasing growth factor concentrations compared to the negative control group (Figure 5A). In contrast, the viability was lower with higher concentrations in the EGF group. At time point 4 days, there was significantly higher viability in the IGF-1 100 ng/mL group compared to the negative control group.

Combining the growth factors KGF and IGF-1 led to an increase in the viability compared to the stimulation with the single growth factors (Figure 5B), which became significant on day 4 compared to KGF.

In nearly all patients, while not reaching statistical significance, the keratinocytes cultivated with the patient-matched ADSC-CM showed higher viability in both the 2-fold and 3-fold concentrated CM groups compared to the negative control groups (Figure 6). There were also high-responder (marked as a square and circle) and low-responder patients with notably above- or below-average stimulation.

**Figure 7:**
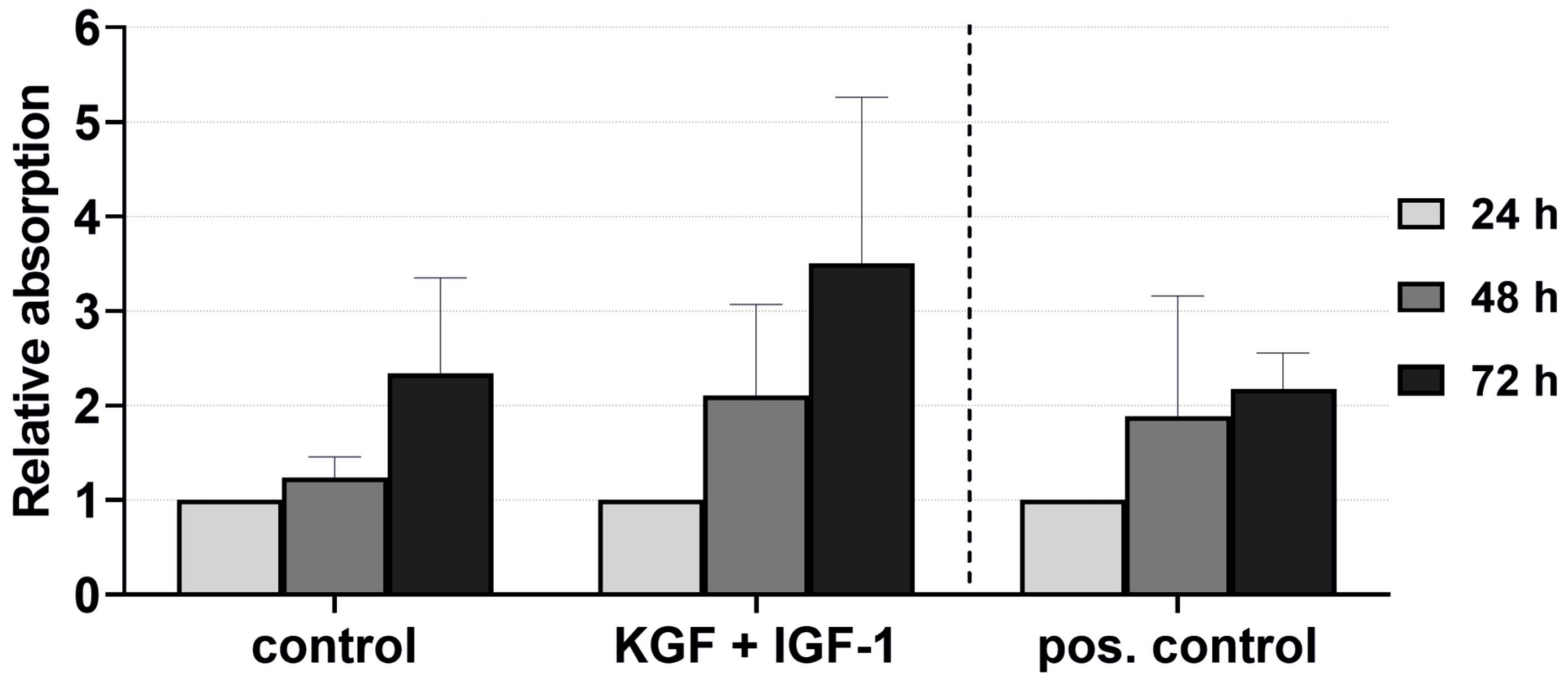
Effect of the growth factors KGF and IGF-1 at a concentration of 100 ng/mL in the xenogen-free medium on the keratinocyte viability immediately after isolation, measured at 24, 48, and 72 h, with absorbance at 24 h = 1. The mean values are plotted, with error bars indicating standard deviation. Negative control: reduced medium; positive control: complete medium (*n* = 5).

The viability of the keratinocytes directly isolated and cultivated in the xenogen-free medium supplemented with KGF and IGF-1 at a concentration of 100 ng/mL was quantified after 24, 48, and 72 h (Figure 7). The keratinocytes showed visibly higher viability with the added growth factors compared to the negative control and even the positive control groups, although these differences were not statistically significant.

### 3.4 Thymosin beta-4 had no effect on the keratinocyte migration, viability, and transmigration

**Figure 8:**
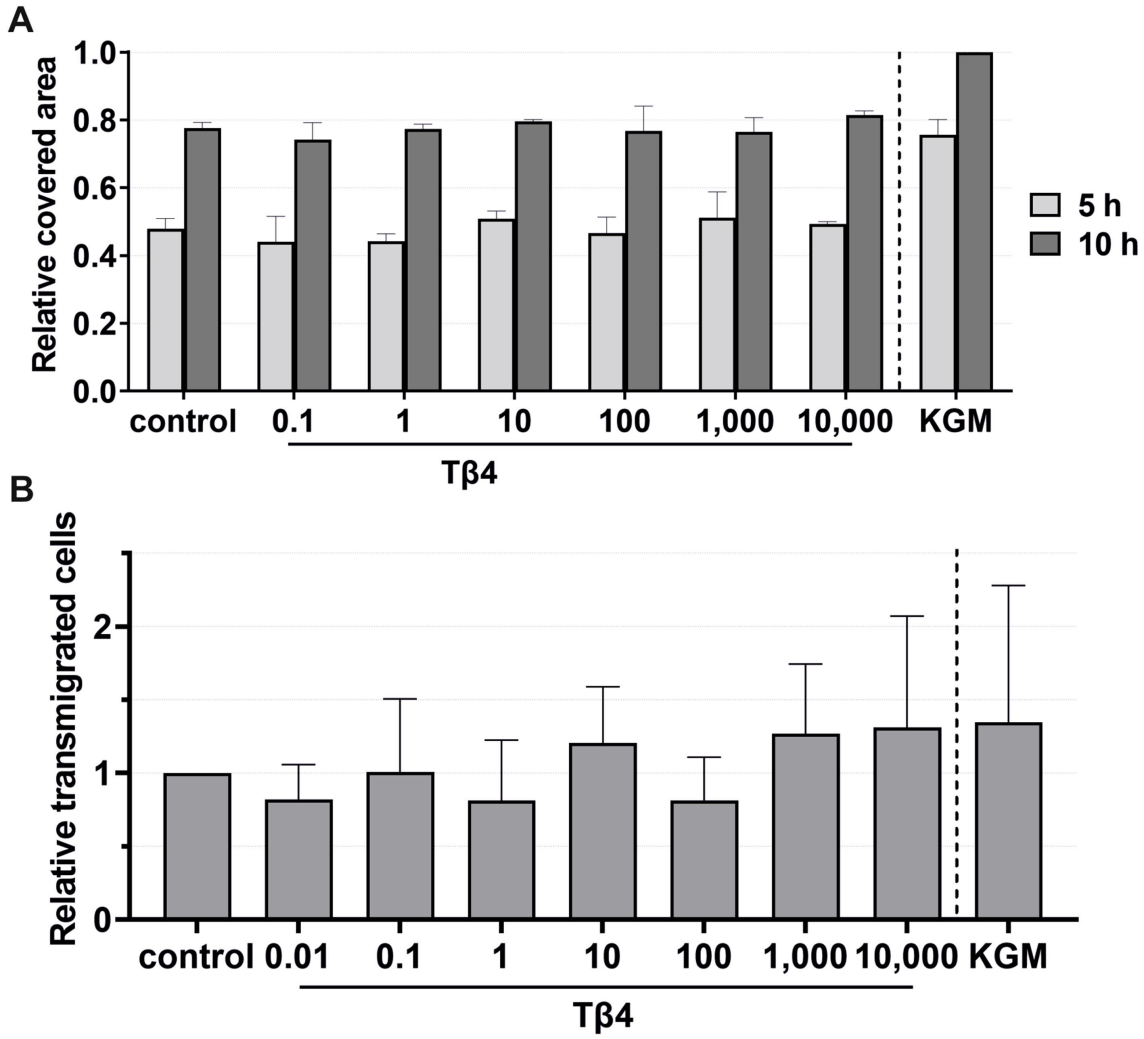
Effects of Tβ4 on the keratinocyte migration and transmigration. The mean values are plotted, with error bars indicating standard deviation. KGM represents the positive control. **(A)** Effects of Tβ4 at concentrations of 0.1, 1, 10, 100, 1,000, and 10,000 ng/mL on the keratinocyte migration. The covered area was measured at 5 and 10 h, with the covered area at 0 h = 0. (*n* = 1). **(B)** Effects of Tβ4 at concentrations of 0.01, 0.1, 1, 10, 100, 1,000, and 10,000 ng/mL on the keratinocyte transmigration after 8 h. The control group was set to 1. (*n* = 4).

**Figure 9:**
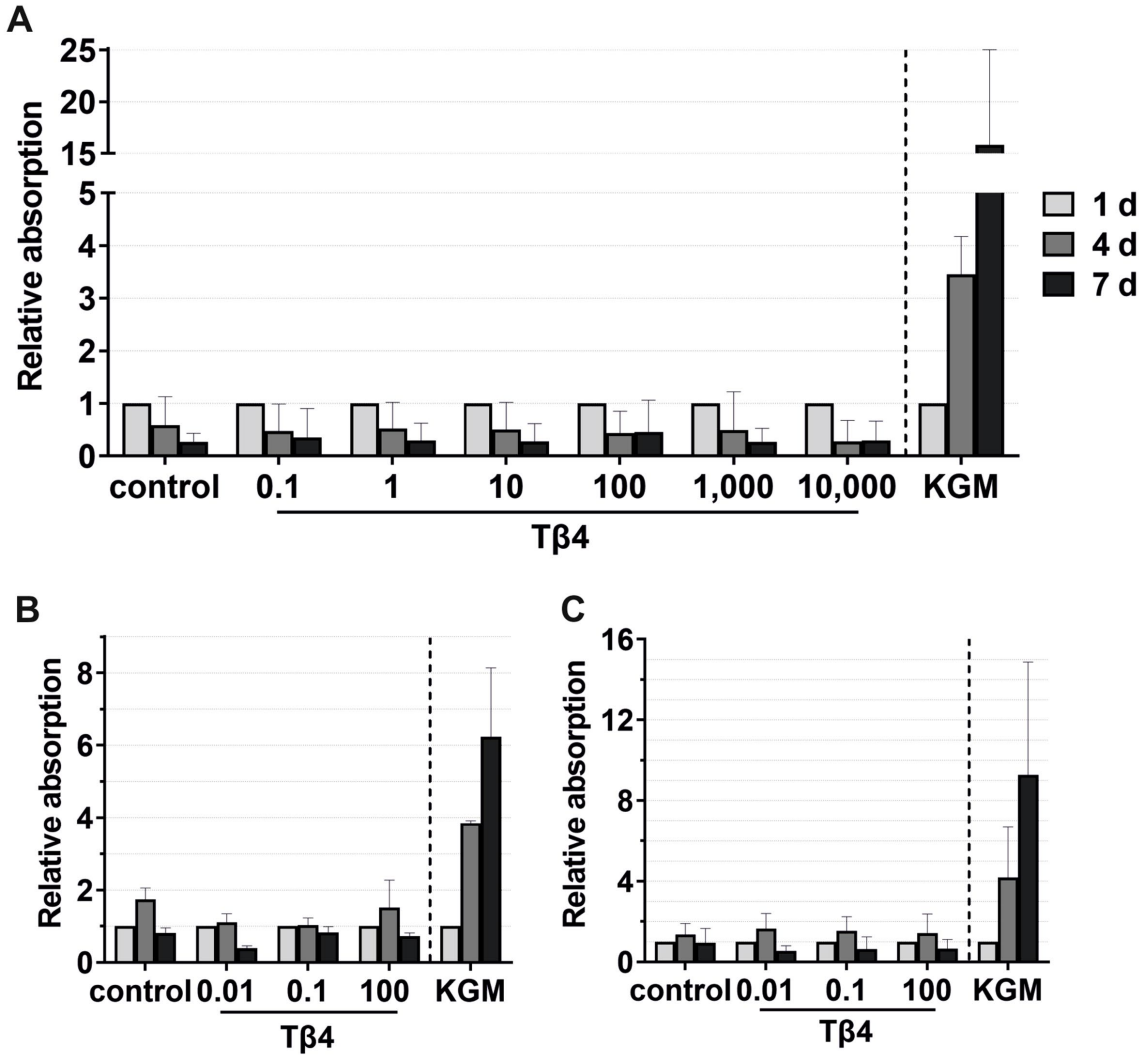
Effects of Tβ4 on the keratinocyte viability. The mean values are plotted, with error bars indicating standard deviation. KGM represents the positive control. **(A)** Effects of Tβ4 at concentrations of 0.1, 1, 10, 100, 1,000, and 10,000 ng/mL on the keratinocyte viability at 1, 4, and 7 days, with absorbance at day 1 set to 1. (*n* = 3). **(B,C)** Comparison of the effects of Tβ4 at concentrations of 0.01, 0.1, and 100 ng/mL in the reduced medium only supplemented with CaCl_2_ **(B)** and the standardized reduced medium **(C)** on the keratinocyte proliferation at 1, 4, and 7 days, with absorbance at day 1 = 1. (*n* = 2).

For migration, viability, and transmigration assays, Tβ4 was used at concentrations of 0.1, 1, 10, 100, 1,000, and 10,000 ng/mL (Figures 8, 9). In all groups, the keratinocytes migrated over time. There was no notable effect on the keratinocyte migration by Tβ4, while stimulation in the positive control (KGM) was possible (Figure 8A). Likewise, the transmigration of the keratinocytes could not be stimulated with Tβ4 (Figure 8B). There was no significant difference in the number of the transmigrated cells compared to the negative control (the absolute average being 25.75 transmigrated cells ±8.71 per ROI) or positive control (the absolute average being 32.22 transmigrated cells ±17.59 per ROI) groups.

For viability assays, Tβ4 was supplemented either to the standardized reduced medium (Figures 9A,C) or to the reduced medium with only the addition of CaCl_2_ (Figure 9B). No significant effect on the keratinocyte viability was observed. In all groups, the cell viability decreased after day 4, while it increased over time in the positive control group (Figure 9A). In the reduced medium with only CaCl_2_, the cells in all groups behaved similarly compared to the negative control group (Figure 9B). Similar results were observed when analyzing the cell viability in the standardized reduced medium with the addition of different Tβ4 concentrations (Figure 9C). In contrast, in the positive control group, the cells showed the expected increase in the viability.

## 4 Discussion

Skin transplantation is of great significance for the treatment of a wide range of diseases. Not only skin injuries but also conditions such as vitiligo are successfully treated with epidermal grafts or keratinocyte and melanocyte cell suspensions (74, 75). A large number of patients with acute or chronic wounds would benefit from the development of methods that promote better healing. In previous studies, the supportive effect of cultured keratinocytes on the healing process of complex wounds has been demonstrated (15). In a diabetic porcine model, Velander et al. (76) demonstrated that an autologous keratinocyte suspension accelerates the healing of full-thickness skin defects. In 2017, Buehrer et al. (77) published a study that assessed the effectiveness of epithelial micrografts in a standardized human wound model with split skin depth. Although there was no difference in healing velocity compared to control sites, more stable wound healing and subjectively softer, more pliable scarring were observed.

Wound healing, in general, is a complex combination of physiological processes involving the interaction of various types of cells, extracellular matrix components, proteinases, and growth factors (78). Growth factors are endogenous signaling molecules that are upregulated in response to tissue damage and are secreted by a variety of cells, such as fibroblasts and epithelial cells. Binding to their respective receptors through autocrine, paracrine, or endocrine mechanisms leads to increases in cell migration, proliferation, and differentiation (79). There is significant interest in establishing growth factor treatments for wound healing. One promising approach is the topical administration of growth factors after surgical debridement (80). Several studies have focused on growth factor delivery systems in the form of particulate systems, scaffolds, hydrogels, and others, which offer promising results and a lot of room for future improvement in the clinical use of growth factors (79).

This study specifically aimed to identify novel approaches for the treatment of epidermal wounds, focusing on the promising future clinical use of keratinocyte suspensions or epidermal grafts. For this purpose, the wound healing properties under different conditions in an *in vitro* model of the human epidermis, using primary cells from various patients, were analyzed. Conditions were defined by the growth factors EGF, IGF-1, and KGF, as well as the peptide thymosin beta-4 at different concentrations and the use of the ADSC-CM. With special attention to the possibility of future clinical application of the findings, the cultivation of keratinocytes in a xenogen-free medium was successfully carried out.

To evaluate possible interindividual differences in wound healing properties between the patients, primary human keratinocytes and conditioned media from the patient-matched ADSCs were used for the present study. The human keratinocytes and matching ADSCs were successfully isolated from the tissue samples obtained from the patients who had undergone body contouring surgery in the abdominal, upper thigh, or upper arm regions. The approach of cultivating human keratinocytes and ADSCs from the same tissue (primarily abdominal fat and skin tissue) derived from the same human adult to conduct patient-matched experiments is unique to this study, as it has not been demonstrated before. Both cell types showed typical morphology, and the keratinocytes were cytokeratin-positive.

There are several growth factors that have promising effects on the functional characteristics of keratinocytes involved in wound healing, such as cell migration and proliferation. It has been shown that EGF leads to increased re-epithelialization in a wound model by promoting keratinocyte migration and proliferation (39–41). Clinically, the topical use of EGF increased the healing rate of chronic wounds in a phase III clinical trial by Park et al. (81). In the present study, EGF had only a small and statistically insignificant effect on the keratinocyte migration and no visible effect on the cell viability, even at quite high concentrations. Haase et al. (82) showed that the combination of IGF-1 and EGF stimulates migration more effectively than their individual use since these growth factors have different effects on migration and act complementarily. EGF seems to be more important for the de-differentiation of keratinocytes to the epithelial linage and for re-establishing the epithelial barrier than for keratinocyte migration (83). Cell migration is prominently stimulated by other growth factors from the EGF family, such as TGF-α, especially in combination with insulin (84). Evaluating the effect of TGF-α or a combination of growth factors from the EGF family on keratinocyte migration could be of interest for further studies. All EGF ligands are synthesized as membrane-anchored forms and must be proteolytically processed to become bioactive soluble factors (85). In the natural environment, both soluble and matrix-bound EGF can be found. Notably, immobilized EGF seems to play a crucial role in single-cell migration (86). One possibility for a better migratory effect could be the usage of photo-immobilized or stabilized EGF (87, 88). In contrast, keratinocyte proliferation might be more effectively stimulated by soluble EGF than by immobilized EGF (89).

IGF-1 is a growth factor believed to play a role in wound healing as its absence, especially in diabetic patients, may lead to delayed wound healing (90). It was reported that deficits in tissue repair in diabetic rats could be reversed by continuous application of IGF-1 (91). Another study with diabetic and non-diabetic mice also showed the effectiveness of IGF-1 in wound healing (92). In the present experiments, IGF-1 displayed a stronger effect on the keratinocytes at higher concentrations, although most of the effects did not reach statistical significance. Statistical significance was only observed in the viability experiments at the 4-day time point with the IGF-1 concentration of 100 ng/mL. Interestingly, some results varied noticeably between the different patients. It is known that IGF-1 and IGF-1R expressions are drastically downregulated in diabetic epidermis, leading to reduced wound-healing capacity (90). All patients in this study were morbidly obese, as high-grade obesity is the main indication for bariatric surgery according to the current German S3 guideline. Several studies have highlighted the high prevalence of high-grade obesity in individuals with type 2 diabetes mellitus (T2D) (93, 94). Therefore, it could be hypothesized that some patients in our study may have been experiencing T2D prior to or during their weight loss, although none of them had been diagnosed with T2D at the time of the body contouring surgery. For the final diagnosis of T2D, insulin release by pancreatic β-cells must be insufficient to fully compensate for decreased insulin sensitivity, leading to glucose intolerance (95, 96). This could explain the patient-dependent differences in IGF-1R expression and the low- and high-responder patients in our study. These findings underscore the importance of therapies specifically tailored to individual patients.

KGF can promote the migration, proliferation, and differentiation of various epithelial cells, including epidermal keratinocytes (53–55). Further, it seems to have a protective effect on damaged epithelial cells (55). In induced wound models of porcine skin, an increased re-epithelialization rate in partial-thickness wounds after the topical application of KGF was observed (97). Several approaches have already been developed for delivering KGF to wounds to support healing processes (56). In line with these findings, in this study, KGF showed stimulatory effects on the keratinocyte migration and viability, with an increasing effect at higher concentrations, although none of the effects reached statistical significance. Combining the two growth factors IGF-1 and KGF led to a notable increase in the cell migration and viability, although the mean values did not reach statistical significance, most likely due to the highly variable interindividual effects.

The above-mentioned growth factors are also secreted by ADSCs located in the subcutaneous fat tissue, in close proximity to keratinocytes. Several studies have shown the secretion of a wide range of cytokines by ADSCs (98, 99). However, few studies have measured the exact amounts of growth factors in a human ADSC-conditioned medium. and if so, the numbers reported on the one hand vary considerably from study to study and on the other hand also vary within the individual studies resp. in between patients that formed part of the study. Authors have reported approximately 12.5 pg/mL of KGF (100), 60–100 pg/mL of KGF (101), and 0.1–16 pg/mL of KGF (102) in the human ADSC-CM. Some studies measured approximately 50 pg/mL of EGF (103), while others described levels ranging from 0 to 40 pg/mL (102). Data on IGF concentrations showed values ranging from 0 to 1,500 pg/mL (with high interindividual differences, the average being approximately 100 pg/mL) (65) or approximately 450 pg/mL (102). Various studies have demonstrated promising results regarding the application of ADSCs for wound healing. ADSCs can be applied directly or their secretome can be used, for example, via conditioned media. Many studies have evaluated the potential effects of mesenchymal stem cells on wound healing.

Human bone marrow-derived mesenchymal stromal cells (BMSCs) were seeded on collagen membranes for transplantation onto cutaneous wounds in mice, leading to faster wound healing and an increase in endothelial progenitor cells and growth factors in the wound (104). Similarly, Luo et al. (105) treated cutaneous wounds in an animal model with co-transplantation of microskin and ADSCs, which resulted in better epithelialization, thinner scars, and increased angiogenesis in the subcutaneous layer compared to control groups. In the clinical setting, mesenchymal stem cells from adipose tissue should be preferably used since they can be more easily harvested from liposuction aspirate or during reconstructive or bariatric surgery compared to BMSCs. Furthermore, approximately 40 to 50 times more ADSCs per gram of fatty tissue can be isolated compared to BMSCs (106, 107). An ADSC-CM could be a promising source for wound healing purposes in the clinical setting since it can be stored in liquid nitrogen (at −196°C) until further use (108) or even lyophilized for simple application at a higher concentration (109). Cultivating ADSCs in xenogen-free platelet lysate, as an alternative to fetal bovine serum-containing media, showed promising results regarding the paracrine effects of the ADSC secretome on keratinocytes in wound healing models (110). In the present study, the ADSC-CM exhibited a remarkable stimulating effect on the keratinocyte migration and viability, similar to the incubation in KGM, although these measurements did not reach statistical significance. However, interindividual differences were again observed, leading to variations in the effectiveness of the ADSC-CM.

Based on the strongly varying concentrations of growth factors in ADSC-CM reported in other studies, we can conclude that there are significant interindividual differences in the growth factor concentrations of individual ADSC-CM, which could explain the patient-dependent variability in the outcomes of the experiments in this study. It could be an interesting approach to experimentally compare the individual secretome with *in vitro* results from wound healing experiments. Schmitz et al. (111) observed highly differing, patient-dependent functional properties in human ADSCs; however, secretome compounds did not correlate with these differences. Instead, the authors found that factors such as sex, lifestyle changes related to exercise or diet, and especially the amount of weight lost appeared to be important. In earlier studies, results concerning the above-mentioned variables varied. Although accumulating evidence has linked factors such as increasing age, body mass index, and diabetes mellitus to a decrease in the functional potential of ADSCs, these effects were not observed in all studies (112). This suggests that more studies with higher numbers of patients are needed.

Due to the small patient number in this study, we could not draw any conclusion about whether the differing effects of the growth factors and ADSC-CM were based on age, gender, BMI reduction, or any other factors. Further studies are necessary to define individual characteristics that lead to the varying stimulation effects of growth factors and ADSC-CM. Nevertheless, based on our results, we can conclude that IGF-1 or a combination of IGF-1 and KGF has the potential to support wound repair and could be useful as a therapeutic tool in wound healing therapies.

The thymus- or platelet-derived protein Tβ4 can be found in wound fluids. It was hypothesized that it supports wound healing. This study aimed to evaluate its effects on a cellular level. Malinda et al. (61) observed a dose-dependent, biphasic increase in the transmigration of mouse keratinocytes *in vitro*. Stimulating effects on wound healing, angiogenesis, and hair follicle development in rodents were also shown by Philp et al. (113). In clinical trials, Tβ4 significantly accelerated wound healing in patients with stasis and pressure ulcers (114). Interestingly, in our study, Tβ4 did not show any effects on the viability, migration, and transmigration of the keratinocytes, although experimental settings were repeatedly adapted. Except for studies using human corneal keratinocytes (115, 116) or human conjunctival keratinocytes (115), most previous studies either focused on rat or mice keratinocytes (61) or were performed in an animal *in vivo* setting (60, 117), making comparisons with our study difficult. To the best of our knowledge, no studies have been published evaluating the effect of Tβ4 on primary human epidermal keratinocytes. There may be significant differences between the response of human keratinocytes and those of other mammals. It is also possible that the lack of significant effects in this study was due to the low number of patients, particularly considering the interindividual differences observed between the patients.

As the growth medium KGM used for the cultivation of keratinocytes contains xenogenic BPE, it would not be suitable for clinical use in humans. In this study, the keratinocytes were directly isolated and cultivated in a xenogen-free medium supplemented with the growth factors KGF and IGF-1. If high numbers of keratinocytes are needed for wound treatment, it would be possible to isolate and culture keratinocytes under GLP principles in such a medium before transplantation. For instance, split skin or epidermal grafts could be harvested and keratinocytes could be isolated, cultured, and directly transplanted to the recipient wounds with combined growth factor supplementation and/or an ADSC-CM. Studies have shown promising effects of autologous keratinocyte injections on full-thickness wounds in *in vivo* models (76). As an alternative, epidermal grafts, as described by Osborne et al. (20), could be directly transplanted to the wound. Combining them with growth factors and/or an ADSC-CM could most probably accelerate wound healing.

Since we observed significant patient-dependent differences in the small group of participants, which could lead to varying responses to this therapy, it is of utmost importance to conduct further studies on this topic. *In vitro* studies with a larger patient cohort and *in vivo* studies using animal models are recommended to assess whether these findings can be validated and translated into clinical trials and eventually into general clinical use.

## 5 Conclusion

In this study, stimulating effects, although not statistically significant, on the keratinocyte migration and viability under the influence of the growth factors, especially the combination of IGF-1 and KGF, and ADSC-CM were observed. The isolation and cultivation of the keratinocytes in a xenogen-free medium with the growth factors IGF-1 and KGF showed promising results. The insights from the present study provide a valuable approach in the field of wound healing and epidermal transplantation. Epidermal grafts or cell suspensions of keratinocytes isolated and cultured under xenogen-free conditions could be combined with these growth factors or an ADSC-CM to accelerate chronic wound healing, helping patients return to everyday life in a shorter time.

## Data Availability

The raw data supporting the conclusions of this article will be made available by the authors, without undue reservation.
